# Case of Supposed Fatal Carditis

**Published:** 1830-01-01

**Authors:** 


### Case of supposed Fatal Carditis. By Mr. Corbin, M.R.C.S and House Surgeon to the Winchester Hospital.*

The following is an extremely interesting case, and may give rise perchance to some differences of opinion. It is so short that we shall give it in the words of the intelligent narrator, and subjoin the few remarks that he has added.

---

* Prov. Med. Gaz. No. II. July.

"The subject of this case was a female servant, ætat. 33, short and unusually muscular. From her statement, she had enjoyed good health for many years past, not being subject to rheumatism, or any other complaint. About eight weeks since, she was attacked with pain in the præcordia, which, though violent from the commencement, and continuing so for the period of three days, did not compel her to apply to any one for relief; but, from the urgency of the pain, was obliged to desist from her work, and keep at rest; at the expiration of this time she suddenly became free from pain, and felt so well, that she now resumed her former employment, and continued in good health till the 6th of February, 1829, when she was again suddenly attacked, whilst washing, with a return of her former pain, the severity of which urged her at once to seek relief, and on her application to the hospital, it was found to be accompanied with the most distressing dyspnœa, dry cough, anxious countenance, violent palpitation of the heart, and a frequent, full, and peculiar jerking pulse. She was immediately bled to syncope, blistered, and the powder of colchicum, in four grain doses, ordered every six hours, from which means she obtained great relief to all her distressing symptoms. On the 11th, the symptoms being still present, though in a subdued form, she obtained a recommendation, and was admitted an in-patient under the care of Dr. Crawford, who ordered her to be bled; but as it was found that the catamenia were at that time flowing freely, the bleeding was postponed. The next morning, February 12, she was found lying tranquilly on the right side, free from pain, but still labouring under dyspnœa, with a very frequent and jerking, though regular pulse. A saline draught, with ten minims of tincture of digitalis, was ordered every six hours, and a purgative draught to be taken immediately. On the following morning she continued free from pain, the catamenia still present, but about midnight all her former symptoms returned, and, on visiting her the next morning, February 14, she was found crouching on her knees in bed, with her head drawn down between her thighs, complaining of great pain all over the left side of her chest, and the pulse beating from 140 to 150 in the minute, full and jerking. The catamenia ceased a little before the recommencement of her pain. She could not lie down in bed in any position, without considerable uneasiness; she was ordered to be cupped immediately, and blistered, and to be bled from the arm, if not soon relieved. Two grains of the submuriate of mercury, with half a grain of opium, were also ordered to be taken every four hours; but death closed the melancholy scene before these remedies were tried.

"*The Dissection* took place about forty hours after her dissolution. On removing the sternum, old adhesions were found in various parts between the pleura pulmonalis and costalis, and about a pint of limpid serum was effused in each cavity of the chest; the lungs, though compressed, contained some bloody and frothy mucus, but were quite healthy in their structure. There was no unusual quantity of fluid in the pericardium, but in patches it was most minutely injected, giving it the most beautiful arborescent appearance. There was neither hypertrophy nor attenuation of the heart, but it was rather larger and redder than usual, and so *exceedingly softened*, that its parietes, as well as the carneæ columnæ, could, without the *least difficulty*, be broken down between the finger and thumb. The semilunar valves, at the commencement of the aorta, were thickened and partly cartilaginous, and the internal surface of the arch of the aorta corrugated, and its coats at this part thickened. The stomach, liver, pancreas, spleen, and intestines, were healthy, as also the kidneys.

"*Remarks.*—It appeared pretty plain, from the remarkable symptoms observed in the above case, that this poor woman's sufferings were to be attributed to some disease of the heart, or of its investing membrane; and, from the morbid appearances found on dissection, I believe it to be a case of acute idiopathic carditis; and I am also led to consider the softening and friability the consequence of inflammation seated in the muscular substance of this organ. It is difficult to distinguish, in practice, between inflammation of the pericardium, and inflammation of the substance of the heart; fortunately, however, it is of no practical importance; though, had the inflammation originally been situated on the pericardium, in this case, from the acuteness of the attack, there would, in all probability, have been effusion of lymph into its cavity, or adhesion to the heart. This highly dangerous affection occurs most frequently in connection with acute rheumatism, but it may also supervene upon any other febrile attack, or it may come on, as in the above case, in an idiopathic form, without any previous disorder."

Whether the above was really a case of carditis, or inflammation of the substance of the heart, we do not profess ourselves competent to decide. The preternatural bulk and redness of the organ are certainly appearances that might reasonably enough be referred to inflammation, but what shall we say to the softening? Laennec, who particularly describes this condition of the heart, does *not* regard it as the sequence of inflammation, and cites some very powerful arguments in favour of his opinion. We recommend our readers to peruse the chapter in Laennec's work on this subject, and we think that when they have done so, they will find their belief, if they had such, in the inflammatory origin of softening to be somewhat shaken.* We do not profess to account for the pain experienced in the præcordia, but we venture to say that it is neither essential to the existence of carditis, nor that of carditis to it. Every practitioner must have witnessed cases of the most severe pericarditis unaccompanied with pain, strictly speaking, though an almost inexpressible anguish generally accompanies the acute cases. In the present instance the effusion into the pleuræ and the state of the lungs are pretty good indices that disease of the heart was going on independent of any occasional inflammatory attacks that might be kindled up.

* Softening of the substance of the heart is not enumerated by Dr. Baillie among the anatomical characters of carditis.—*Rev.*

